# Understanding social resilience in honeybee colonies

**DOI:** 10.1016/j.cris.2021.100021

**Published:** 2021-10-23

**Authors:** Zeynep N. Ulgezen, Coby van Dooremalen, Frank van Langevelde

**Affiliations:** a Bees@wur, Wageningen University and Research Centre, Droevendaalsesteeg 1, Wageningen 6708 PB, The Netherlands; b Wildlife Ecology and Conservation Group, Wageningen University and Research Centre, Droevendaalsesteeg 3a, 6708 PB Wageningen, The Netherlands; c School of Life Sciences, Westville Campus, University of KwaZulu-Natal, Durban 4000, South Africa

**Keywords:** coping mechanism, division of labor, thermoregulation, timing of brood rearing, colony losses, stressor

## Abstract

Honeybee colonies experience high losses, induced by several stressors that can result in the collapse of colonies. Experiments show what effects stressors, such as parasites, pathogens and pesticides, can have on individual honeybees as well as colonies. Although individuals may die, colonies do not always collapse from such disturbances. As a superorganism, the colony can maintain or return back to homeostasis through colony mechanisms. This capacity is defined as social resilience. When the colony faces a high stress load, this may lead to breakdown in mechanisms, loss in resilience and eventually colony collapse. Before social resilience can be measured in honeybees, we need to examine the mechanisms in colonies that allow recovery and maintenance after stressor exposure. Here, we discuss some of these mechanisms and how they affect the social resilience of honeybee colonies. Understanding social resilience in honeybees is essential to managing colony health and loss prevention.

## Highlights

• Social resilience allows honeybee colonies to maintain and recover to homeostasis
• Loss of social resilience from exposure to stressors can lead to failure of colonies
• There is a lack in understanding of coping mechanisms for social resilience
• Focusing on indicators of resilience in colonies can help prevent colony collapse

## 1 Introduction

Honeybee species form a major part of the pollination service provided by insects (Klein et al., 2007; Hung et al., 2018). In the past several decades, honeybee colonies have dealt with high annual losses, especially in the Northern Hemisphere (Neumann and Carreck, 2010; Kulhanek et al., 2017). Several stressors including parasites, pathogens and pesticides, have been implicated as the causes of colony loss (Potts et al., 2010; Vanbergen and The IP Initiative, 2013; Goulson et al., 2015; Sánchez-Bayo and Wyckhuys, 2019). Not all colonies collapse, however, as colonies have the capacity to recover from these challenges (e.g. Berenbaum and Johnson, 2015; De Smet et al., 2017; Laomettachit et al., 2021)

Preventing loss of honeybee colonies requires understanding beyond assessing the effects of long-term and short-term stressors on individual bees. Eusocial insects (i.e. ants, bees, wasps and termites) are considered to be superorganisms, defined as an aggregation of individuals that function as an integrated whole (Seeley, 1989). Analogous to germ and somatic cells in single multicellular organisms, a few individuals monopolize reproduction in superorganisms and the rest perform other life sustaining functions. The loss of workers can be tolerated as long as survival (important for an individual colony) and reproductive capacity (important for the population of colonies) are conserved (Cremer et al., 2018). The capacity of superorganisms to firstly maintain homeostasis and to secondly recover from and return back to homeostasis, is defined as social resilience (Sendova-Franks and Franks, 1994; van Dooremalen et al., 2018). For instance, social immunity in honeybees, where colonies possess several traits to combat and protect against pathogens, parasites and pesticides (Simone-Finstrom, 2017), is a form of social resilience. Colonies can have traits, such as corpse removal (van Langevelde et al., 2020), hygienic behaviour (Panziera et al., 2017), grooming (Kruitwagen et al., 2017) or the recently discovered suppressed *in ovo* virus infection (de Graaf et al., 2020) that can make honeybees more resilient against pathogens and parasites on a colony level. It is important to do stress exposure experiments on colony level as a recent study shows that when bees were exposed to sublethal doses of the pesticide imidacloprid, honeybees in cages showed immune suppression while honeybees in colonies showed immune stimulation (De Smet et al., 2017). Hence, understanding social resilience is essential for managing the health of honeybee colonies.

Progress in technology and development of novel analytical tools has opened up possibilities to find indicators of loss of resilience (Scheffer et al., 2018). Yet, before these methods can be applied to prevent colony loss, it is important to address the question what general behavioural mechanisms can be found in honeybee colonies that help maintain homeostasis and allow colonies to recover from stress? Here, we discuss behavioural mechanisms of honeybees for social resilience and how these mechanisms may be affected by stressors.

## 2 Homeostasis and social resilience

Homeostasis is fundamental for all living organisms and entails the regulation of balanced internal states for maximizing fitness (Calow, 1982). Over evolutionary time, organisms are thought to have evolved different strategies to maintain and return to homeostasis in a world of unpredictable perturbations. Maintenance of homeostasis may occur in honeybees passively through stress tolerance, such as colonies surviving infestation of the parasitic mite *Varroa destructor* without treatment (Locke, 2016), or actively through resistance, such as colonies surviving through cold winters by actively thermoregulating hive temperature (Southwick, 1985). Colonies may also show fast adaptation to, or recovery after stress, through behaviours like recruitment of foragers after high losses (Johnson 2010; Perry et al., 2015). Maintenance and recovery trade-offs might be a commonality across different systems in describing mechanisms of resilience exposed to perturbations (Côté and Darling, 2010; Hodgson et al., 2015), such as in eusocial insects. In superorganisms such as eusocial insects, there is a dual challenge of coping with disturbances; in addition to adaptations at the individual level, nestmates must adapt their collective behavioural strategies to cope with these changes together (Schmickl and Crailsheim, 2004). High plasticity of such coping mechanisms in honeybee colonies may also increase their social resilience in response to stress (Straub et al., 2015; van Dooremalen et al., 2018), similarly as occurs in ant colonies (Sendova-Franks and Franks, 1994).

**Figure 1:**
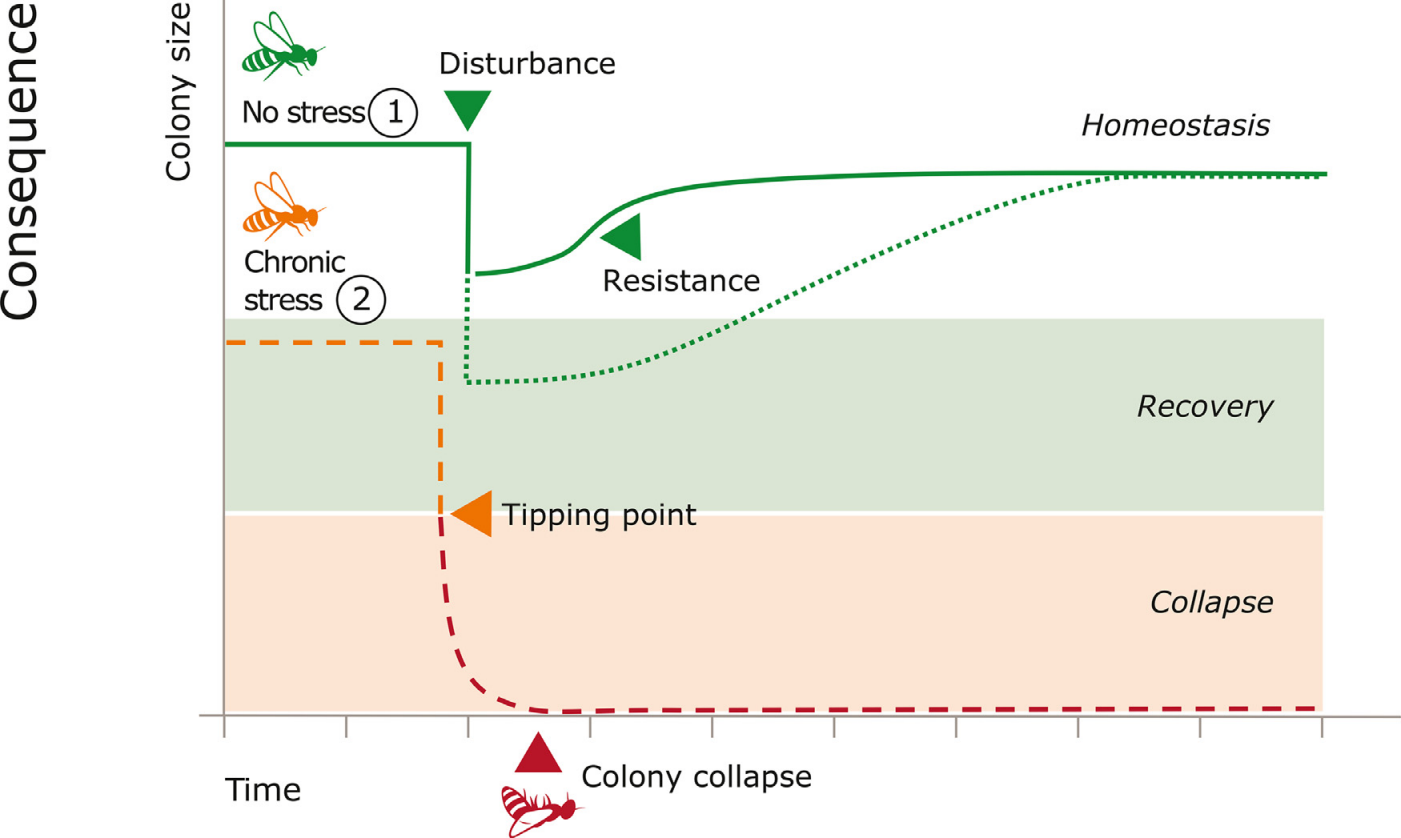
Hypothetical honeybee colony size after a disturbance in chronically stressed and healthy colonies. Healthy colonies (1) show resistance to maintain homeostasis or recover back to homeostasis after stressor exposure. Colonies under chronic stress (2) (e.g., the parasitic mite *V. destructor*) are more vulnerable to the effects of the disturbance, they can be pushed past the tipping point and may be unable to recover.

Social resilience in honeybees can become compromised due to chronic stress, such as exposure to the parasitic mite *V. destructor* (van Dooremalen et al., 2018). When stress is sustained for too long, the cost of maintaining or recovering to homeostasis can lead to the gradual depletion of resilience (Romero et al., 2009). As an organism becomes less resilient, it becomes more sensitive to environmental fluctuations and perturbations (Dakos et al., 2010). The loss of resilience may propel the organism to a tipping point, where after a certain threshold, a critical transition occurs to a contrasting state (van Nes et al., 2016). At this state, the organism may be unable to cope with disturbances, and minor perturbations can lead to its collapse (Scheffer et al., 2018) (Fig. 1). Recent theoretical studies support the idea that loss of resilience can trigger collapse in honeybee colonies (Perry et al., 2015; Bastiaansen et al., 2020). Therefore, it is important to understand how stressors act on social resilience.

## 3 Coping mechanisms in honeybee colonies

The annual lifecycle of honeybee colonies in temperate regions involves three important stages. (1) During winter, ambient temperatures are low and there are limited resources. For survival, the worker bees in the colony cease foraging and brood rearing activities (Seeley and Visscher, 1985; Southwick, 1991), and form a thermoregulatory cluster to reduce heat loss (Southwick, 1985). All winter bees are generalists, they perform common tasks, such as generating heat by consuming stored resources. (2) By the end of winter, winter bees in colonies resume brood rearing in anticipation of foraging and resource exploitation in spring. To produce future work force, colonies start to rear brood during late winter, by further use of stored resources. (3) After nest emergence in spring and throughout summer, honeybees use the window of abundant resources in the environment for rapid colony growth and reproduction (swarming), and store food in preparation for winter. To achieve this, summer worker bees perform a multitude of tasks, where they segregate and change roles in the colony with age (Robinson, 1992). The specialization and parallelization of this division of labour (DOL) in summer, optimizes efficiency and hence productivity of the colony (Johnson, 2010). The underlying mechanisms involved to facilitate these stages enable the colony to be highly adaptive to a variable environment.

Honeybee colonies exhibit behavioural plasticity, where behaviour of individual bees can be altered due to environmental conditions and colony demography, in order to meet colony demands accordingly (e.g. Huang and Robinson, 1992; Jones and Oldroyd, 2006; Matilla and Otis, 2007; Nürnberger et al., 2018). Adhering to the annual life-cycle of colonies, we suggest to describe the underlying coping mechanisms that honeybees utilize for social resilience as: thermoregulation in winter for survival and conservation of resources, timing of brood rearing in spring for future reproduction (swarming) and workforce, and division of labour in summer for resource acquisition. When the stress load (duration x severity) exceeds the resilience capacity of a colony, breakdown or alterations in these three coping mechanisms that enable resilience may lead to high losses (Barron, 2015).

### 3.1 Thermoregulation

In temperate zones, winter is a critical period of survival for honeybee colonies, as mortality is high during this season (Genersch et al., 2010). To survive through low ambient temperatures, colonies form a thermoregulatory cluster (Southwick, 1985) at more or less constant temperature levels, regardless of fluctuations in ambient temperatures (Southwick, 1982). Heat production is self-organized (Watmough and Camazine, 1995), where individual workers start shivering flight muscles when temperature drops below a certain level (Seeley, 1985). Homeostasis, in terms of thermal stability, is maintained by bees at the core of the cluster and insulation by the bees on the periphery (Heinrich, 1981). If a honeybee cools down below a certain threshold level it will experience a chill coma and no longer be able to shiver flight muscles and generate heat (Goller and Esch, 1990). This immobilization may make it unable to search for food in the hive, eventually leading to death by starvation despite the availability of food reserves (Bastiaansen et al., 2020). Therefore, the maintenance of the core temperature of honeybee colonies is important as a coping mechanism for both individual and colony survival.

In response to a drop in ambient temperature, bees in the periphery of the cluster pack tightly, increasing its density and insulation (Watmough and Camazine, 1995). Further decrease in temperature requires the active production of heat. The energy used for heat production by an individual bee becomes higher as the number of honeybees in the cluster decreases (Fahrenholz et al., 1989; Stabentheiner et al., 2003). This triggers a positive feedback as increased work load leads to a shorter life-span of the individual bees and hence to a smaller thermoregulatory cluster (Bastiaansen et al., 2020).

**Figure 2:**
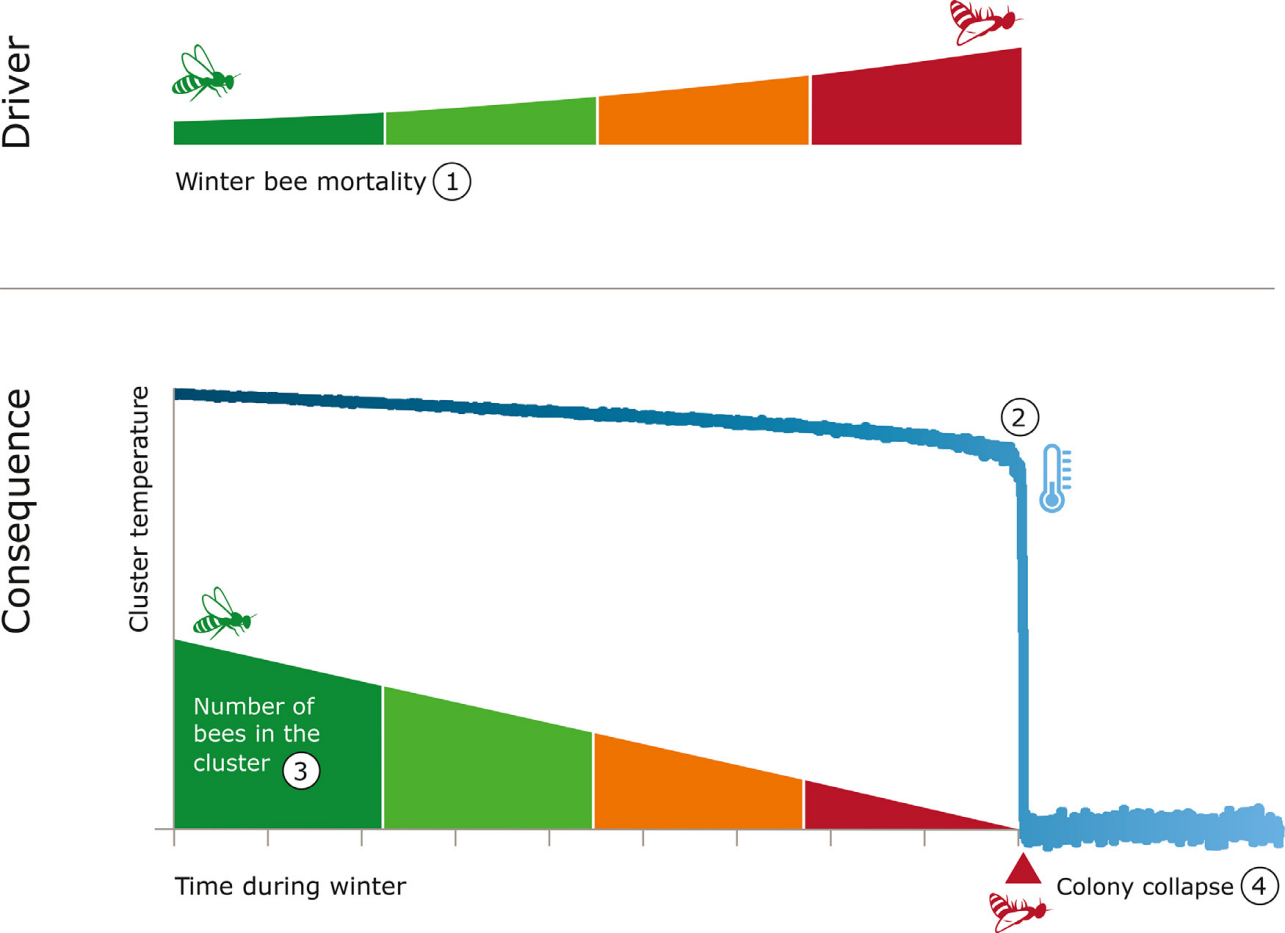
The effects of thermoregulation capacity on the survival of honeybee colonies in winter. Colonies that are chronically stressed may have a higher mortality rate in comparison to healthy colonies. For instance, stressors such as the parasitic mite *V. destructor*, on an individual level, can lead to physiological problems with flight muscles (Blanken et al., 2015). Previous experiments show that *V. destructor* is a vector for diseases like Deformed Wing Virus (Martin et al., 2012), which cause developmental deformations on the wings of bees. This may have consequences on both worker efficiency and mortality (1), leading to insufficient heat generation (2). Secondly, *V. destructor* can reduce colony size (Dainat et al., 2012; van Dooremalen et al., 2012). Thermoregulation capacity may be hindered as a result of smaller cluster size (3) and inefficient workers. This in turn may have consequences on cluster temperature, and over time may lead to colony collapse (4).

Stressors that have an effect on colony size or worker condition may lead to the failure of thermoregulation as a coping mechanism and ultimately to colony collapse. Pesticide exposure through stored food resources is thought to be a stressor leading to winter mortality. Neonicotinoids can have lethal consequences on winter bees, as they have been found to be more toxic to honeybees in lower temperatures (Saleem et al. 2020) and can reduce winter bee survival (Baines et al. 2017; Wood et al 2020). There are also a variety of sublethal effects of pesticides on worker bees. For instance, honeybees fed the neonicotinoid thiamethoxam had altered thorax temperatures, potentially effecting their thermoregulation capacity (Tosi et al., 2016). Furthermore, high infection levels with the stressor *V. destructor* may reduce the lifespan of winter bees, leading to smaller colonies prior to winter (van Dooremalen and van Langevelde 2021). Such stressors can have an impact on thermoregulation through its effects on the performance of individual bees and colony size, and push a colony out of homoeostasis. Bastiaansen et al. (2020), by modelling winter colony survival, suggest that when colony size is too low and the core temperatures of a colony drops below a critical threshold a rapid decrease in bee population occurs and sudden death of colony is predicted (Fig. 2).

### 3.2 Timing of brood rearing and nest emergence

In spring (after nest emergence), foragers will start mass foraging to replenish pollen, used as protein source for brood rearing in summer, and nectar, used as (stored) energy source for flying in summer and thermoregulation in winter (Seeley and Visscher, 1985). In anticipation of foraging and resource acquisition in spring, colonies start brood rearing in late winter. The changes in overwintering bee colonies, especially the broodless state is an adaptive response to low food availability and critical for winter survival of the honeybee colony (Seeley and Visscher, 1985). Premature brood rearing and nest emergence, in relation to new resource acquisition, can lead to depletion of stored winter resource and nurse bees before they can be replenished. Delaying winter brood rearing can hinder the exploitation of spring bloom and hamper colony growth and timely reproduction.

Cues of seasonal change, namely photoperiod length and temperature, have been suggested to play a role in the onset of brood rearing in honeybee colonies (Nürnberger et al., 2018, 2019). Furthermore, changing pollen stores in a colony, by either feeding supplements or restriction of stores, can respectively delay or accelerate bees going into the wintering state, due to changes in the size of the brood nest (Matilla and Otis, 2007). While further research is necessary to understand how the mechanism behind timing of brood rearing and nest emergence works, these studies suggest that it may be a mechanism to cope with changes in environmental factors and colony resources.

**Figure 3:**
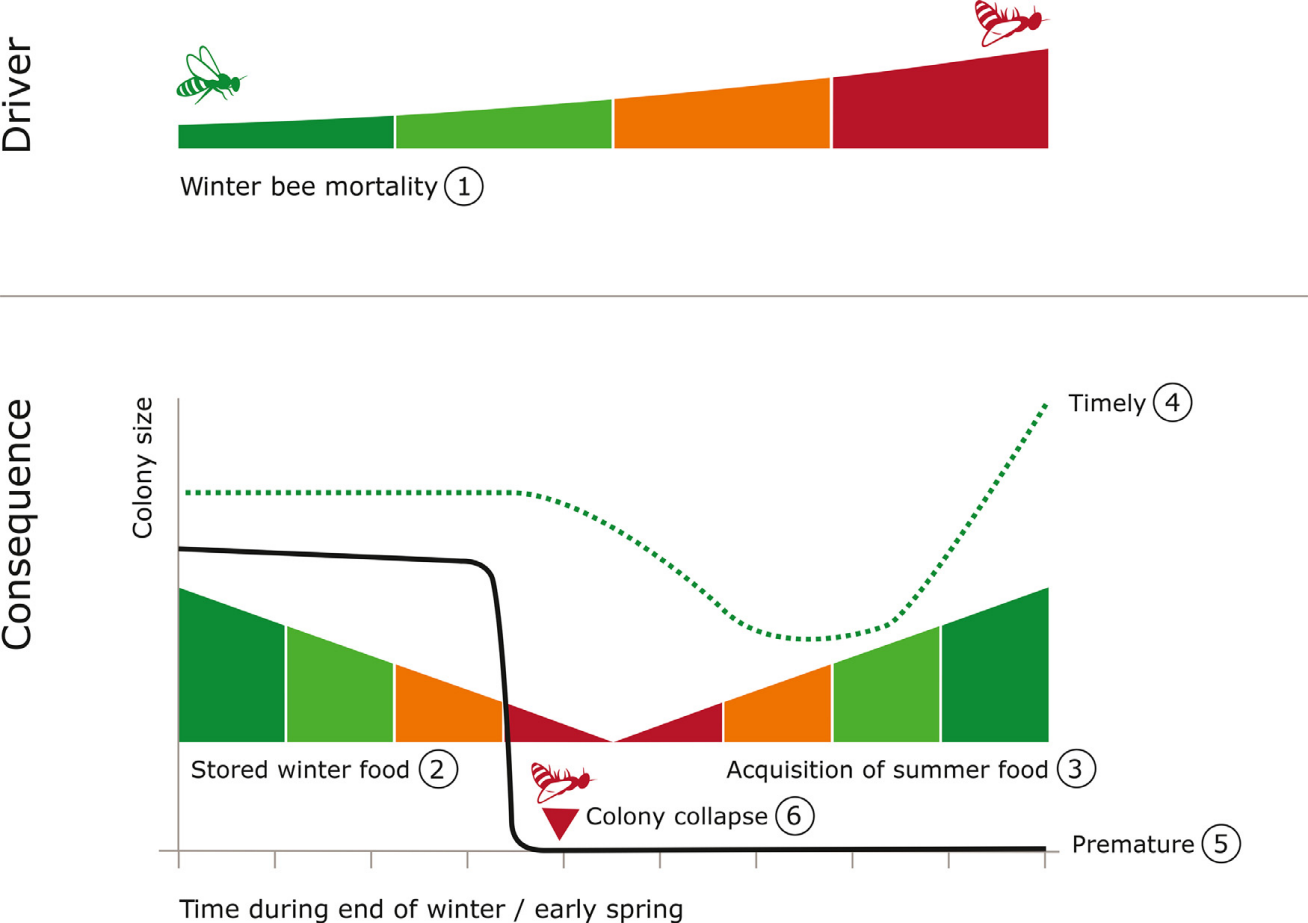
The effect of timing of brood rearing and nest emergence on honeybee colony size from winter till spring. After the wintering period, starting of brood rearing will have costs on colonies, in terms of decreased life-span and higher mortality of bees (1), and greater use of stored food resources (2). Colonies that will start brood rearing will first have an increased energy expenditure. These costs account for the dip in colony size before colony growth in colonies with timely start of brood rearing and nest emergence. Timely colonies will be able to optimally utilize available floral resources (3) to start grow in size (4) and later on reproduce. Chronically stressed colonies may start brood rearing prematurely (5) compared to healthy colonies with non-shifted phenology. Stressors such as *V. destructor* may push colonies into premature brood rearing due to reduced life-span of winter bees (see 1) (Amdam et al., 2004). Early onset of brood rearing and nest emergence may lead to temporal mismatch with floral resources (Schenk et al., 2018) and hinder the acquisition of summer food. In honeybees, the restriction in pollen availability has been shown to reduce brood size (Schmickl and Crailsheim, 2001), which in turn may lead to a smaller colony size over time. Therefore, premature brood rearing may lead to depletion of colony resources and eventually collapse (6).

Habitat loss and low food availability are currently suggested as stressors that cause colony losses (Sánchez-Bayo and Wyckhuys, 2019). Furthermore, climate change, specifically winter conditions, has been found to alter phenology in many terrestrial species, including insects (Williams et al., 2015). Exposure to stressors may cause a shift in colony phenology and lead to colony failure due to temporal mismatches with environmental resources (Fig. 3). A discrepancy between timing of brood rearing and nest emergence and food availability in the environment may lead to resource shortages and malnutrition in the colony. Several issues in colonies may arise, including cannibalism of the younger larvae (Schmickl and Crailsheim, 2001), and physiological deficiencies in worker bees (Crailsheim and Stolberg 1989; Schmickl and Crailsheim, 2001). Such sublethal effects may have a carry-over effect and compromise colony growth, causing failure in colony functions and preparation for winter survival.

### 3.3 Division of labour

DOL in honeybees starts after nest emergence in spring, and is predominantly seen during summer, when there are abundant resources. Typically, workers of honeybee colonies demonstrate DOL known as temporal polyethism, segregating and changing roles in the colony with age (Robinson, 1992). Bees perform several different types of tasks throughout their lifetime, respectively as: cell cleaners, cleaning cells recently evacuated by brood; nurses, primarily tending the brood; middle aged bees (MABs), which have a variety of in-hive tasks, such as storing food, guarding and nest building; and foragers, gathering resources (nectar, pollen, propolis and water) for the colony (Seeley, 1985).

Notably, honeybee colonies show flexibility in DOL, by accelerating, delaying or reversing behavioural and/or physiological development of workers, based on colony demography (Huang and Robinson, 1996), foraging activity and pollen availability (Fewell and Winston, 1992) to meet colony needs. It is proposed that the balance of DOL within a colony is controlled by a combination of a social inhibition mechanism that freezes a worker in a task, and environmental triggers, which release a worker from that task (Johnson, 2010). For instance, there is a negative feedback mechanism between proportion of foragers and recruitment of younger bees to the foraging task, where pheromones of foragers, specifically ethyl oleate, passed onto other bees through trophallaxis, inhibit the recruitment of bees (Leoncini et al., 2004). In cases where there is an increase in availability of pollen, rate of contact between foragers and food receivers decrease, leading to the increase of foragers in the colony (Johnson, 2010). Therefore, honeybees use DOL as a coping mechanism to environmental changes.

**Figure 4:**
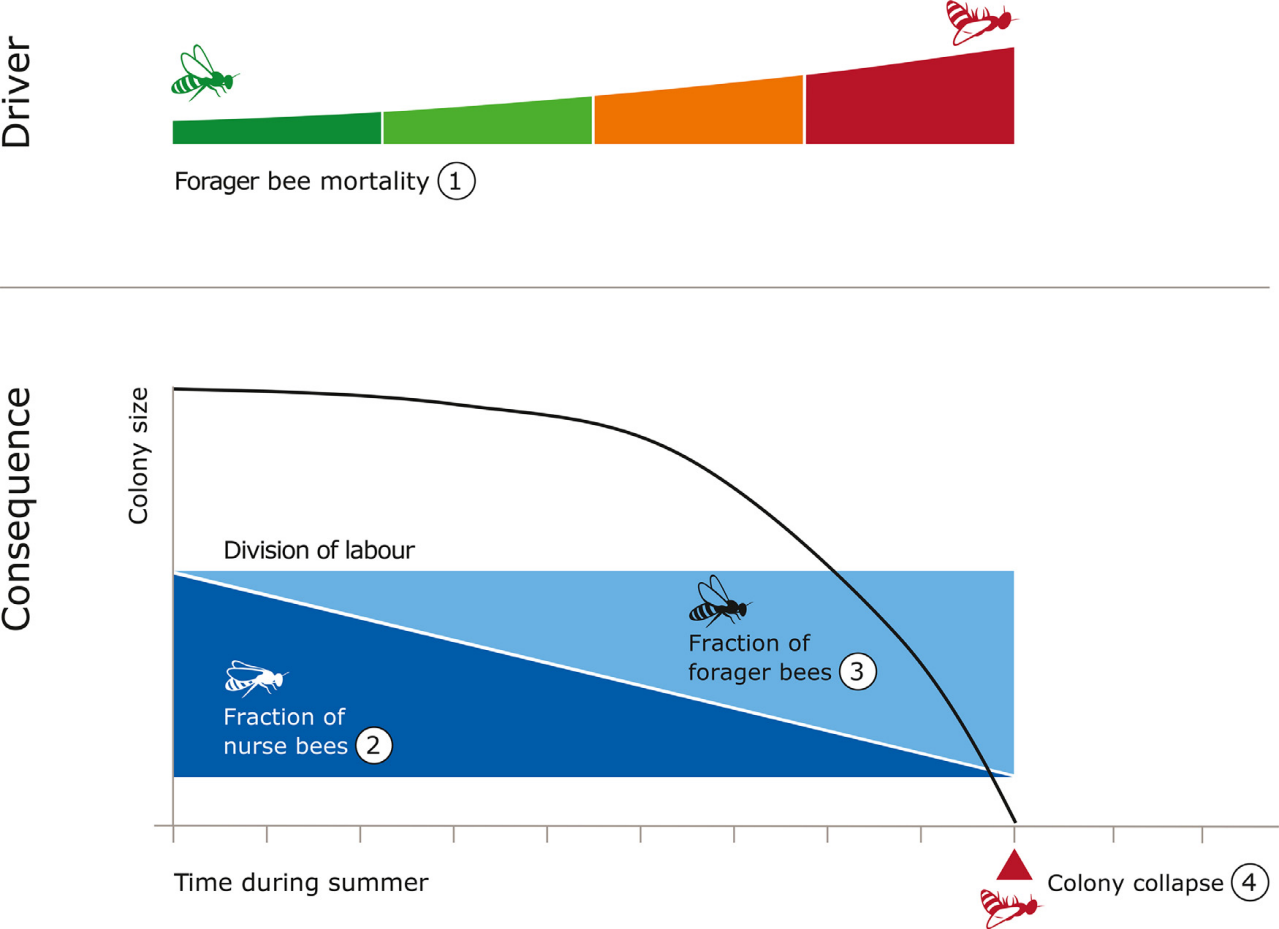
The effect of shift in distribution of division of labour in summer on honey bee colonies. Colonies under stress may experience higher forager bee mortality (1), for instance due to pesticide exposure, compared to healthy colonies. Loss of foragers lead to early recruitment of young bees to the foraging task, where these young bees are less effective foragers and experience a higher risk of mortality compared to bees that start foraging at a later age (Perry et al., 2015). To compensate from these exacerbating circumstances of forager inefficiency and losses, over time, colonies may increase recruitment rate of forager bees at an earlier age, shifting demography from nurses (2) to more foragers (3). This can create a positive feedback, potentially depleting or reducing the capacity of resource allocation in the colony, eventually causing collapse (4).

Changes in the distribution of workers and worker efficiency due to stressor exposure might lead to the failure of DOL as a coping mechanism (Fig. 4). For instance, the microsporidian parasite *Nosema spp.* leads to a higher mortality in bees (Williams et al., 2014), possibly reducing social inhibition and causing the recruitment of younger bees to the foraging task (Dussaubat et al., 2013). These changes in DOL may be further exacerbated by sublethal effects of the stressor. Nosema-infected bees have been shown to exhibit more stationary behaviour compared to healthy bees (Retschnig et al., 2015). In conjunction with less efficient workers from reduced mobility, this may also lead to issues with social interactions within the colony, as pheromones, important for communication and play a role in task switching, are passed on through contact (Johnson et al., 2010). Thus, stressors that cause high mortality, alter the social inhibition mechanism and affect the ontogeny of worker behaviour can cause a shift in DOL, compromising social resilience. Several theoretical studies show that there may be a shift in colony demography towards precocious foragers when exposed to stressors, which may lead to a rapid loss of workers and eventually colony collapse (Barron, 2015; Perry et al., 2015).

## 4 Implications and conclusions

We argue that there is a need to study the functioning of the three identified coping mechanisms and what changes occur in response to stress. Breakdown or alterations in these mechanisms can be identified by looking at colony level traits. For instance, colony size relates to the functioning of thermoregulation in winter and infestation with *V. destructor* may lead to decreases in colony size (Dainat et al., 2012; van Dooremalen et al., 2012), which may reduce thermoregulation capacity. To test whether this implies a loss of resilience, we propose to expose both healthy and stressed colonies to an environmental disturbance and monitor whether it triggers the stressed colonies towards a tipping point compared to the healthy colonies (Fig. 1). Colony size reduction, longer return time to homeostasis and/or higher variations in colony traits after disturbance may suggest there is indeed a loss of resilience over time (see the methods for detecting critical transitions in Dakos et al., 2012). This method of experimentation can help in finding indicators of resilience prior to colony collapse. It may be of importance to take the origin of the colonies into account, e.g. there is evidence that locally adapted honeybees have a higher probability of winter survival compared to selection lines, as they are considered to be more resilient against local conditions (Kovačić et al., 2020). Research into resilience mechanisms are not only relevant in the context of loss prevention, but also for future improvement of breeding programs for sustainable beekeeping.

Identifying social resilience in colonies is essential for honeybee health and preventing collapse. Theory predicts that there is a trade-off between the ability to recover, i.e. fast growth under benign conditions, and the ability to resist or tolerate stress (Pianka, 1970; Grime, 1997). Current studies are far from understanding these trade-offs in honeybee colonies. Classical methods of measuring colony health are by visual inspection, where presence of parasites or viruses and, colony traits such as colony size, honey and pollen storage and brood size are recorded (Delaplane et al., 2013). These methods that only give a momentary snapshot of a colony are unsuitable considering the complex and dynamic characteristics of superorganisms (van Dooremalen and van Langevelde, 2021). Recent studies suggest that technological developments that allow for more continuous measurements and emergence of novel analytical tools has provided an opportunity to find indicators of loss of resilience (Scheffer et al., 2018). Measurements from in-hive sensors can allow us to gather high resolution information on colony dynamics (Meikle and Holst, 2015). Studies give emphasis on the use of weight, sound, temperature and vibrations to measure colony resources, activity and growth (Meikle and Holst, 2015; Zacepins et al. 2015). It is beneficial to investigate the opportunities these tools may provide for sustainable management of honeybee colonies.

## CRediT authorship contribution statement

**Zeynep N. Ulgezen:** Writing – original draft, Writing – review & editing. **Coby van Dooremalen:** Writing – original draft, Writing – review & editing. **Frank van Langevelde:** Writing – original draft, Writing – review & editing.

## Declaration of Competing Interest

The authors declare that they have no known competing financial interests or personal relationships that could have appeared to influence the work reported in this paper.
